# The transversus abdominis plane block may reduce chronic postoperative pain one year after TAPP ingunial hernia repair

**DOI:** 10.1016/j.amsu.2020.04.011

**Published:** 2020-05-23

**Authors:** Christoph Paasch, Jennifer Fiebelkorn, Gianluca De Santo, Sascha Azarhoush, Katherina Boettge, Stefan Anders, Ulrich Gauger, Martin Strik

**Affiliations:** a Department of General, Visceral and Cancer Surgery, Helios Klinikum Berlin-Buch, Schwanebecker Chaussee 50, 13125, Berlin, Germany; b No Insurance Surgery, 653 N Town Center Drive, 89144, Las Vegas, United States; c Private Statistical Consultant, Germany

**Keywords:** Transversus abdominis plane, TAP block, Chronic pain, TAPP, Inguinal hernia repair, Chronic postoperative inguinal pain, Pain exertion

## Abstract

**Introduction:**

Chronic postoperative inguinal pain (CPIP) is defined as pain impacting daily activities lasting at least 3 months. With an incidence of 0.5–6.0%, chronic pain affects many patients who underwent inguinal hernia repair (IHR). Early severe postoperative pain has been described as a risk factor for CPIP. Thus, we aim to investigate the impact of the transversus abdominis plane (TAP) block on CPIP.

**Methods:**

From 2013 to 2019 we collected data from individuals who were operated on electively in TAPP technique and who received a preoperative TAP block.

**Results:**

Data from 289 patients were selected. 259 patients were male. The mean age was 59.93 years and the mean BMI was 25.72 kg/m2. 252 patients suffered from a primary inguinal hernia. No mesh fixation was conducted. 21 patients reported pain at rest, 26 pain under physical exertion and 13 patients required treatment of their pain. In 6.25% of cases patients reported CPIP. We compared our findings with data from the German Herniamed Registry (unilateral, primary IH, men, no mesh fixation; n = 8.799), because we assume that the majority of these patients did not receive a TAP block. The rate of pain under physical exertion (9.2% vs. 10.05%) and pain requiring treatment (2.45% vs. 2.95%) one year after surgery slightly differs without a statistical significance.

**Conclusions:**

We assume that the TAP block may reduce CPIP, postoperative pain during physical exertion and pain requiring treatment following IHR in TAPP technique. Additional randomized clinical trials are mandatory to evaluate the hypothesis.

## Highlights

• The TAP block may reduce the rate of chronic postoperative inguinal pain.
• The TAP block may reduce the rate of pain requiring treatment and on exertion one year after IHR.
• The TAP should further implemented into daily hernia surgery routine.

## 1 Introduction

Chronic postoperative inguinal pain (CPIP) is defined as pain impacting daily activities lasting at least 3 months postoperatively [1,2]. With an incidence of 0.5–6.0% chronic pain affects many individuals who underwent open and laparoscopic inguinal hernia repair (IHR) repair [3,4]. Young age, female gender, high preoperative pain, early high postoperative pain, recurrent hernia and open repair has been described as risk factors for CPIP [1].

To reduce the analgesic medication, pain in the Post-Anesthesia Care Unit (PACU) and to accelerate the physical recovery time, the administration of local and regional anesthetic agents has been implemented in parts into the daily routine of hernia surgery. Like it has been recently published by different authors the transversus abdominis plane (TAP) block prior to IHR in TAPP technique reduces the cumulative analgesic need medication and the pain level in the PACU [5]. This approach consists of the administration of local anesthetic in the layer between the internal oblique and transversus abdominis muscles. Target of this procedure are the sensory nerves innervating the abdominal wall originating from T7 to L1 (intercostal, ilioinguinal, subcostal, and iliohypogastric nerves) [[6], [7], [8]].

We hypothesize, that the reduction of early postoperative pain, a CPIP risk factor, after TAP block administration may reduce postoperative painn following IHR in TAPP technique.

## 2 Methods

A monocentric retrospective observational study investigating the TAP block impact on chronic postoperative inguinal pain (CPIP) was conducted. According to International guidelines CPIP was defined as pain impacting daily activities lasting at least 3 months postoperatively [1,2].

The data of individuals who underwent elective IHR in TAPP technique from January 2013 to January 2019 were taken from their electronic files.

The study has been performed at the xxx hospital (Germany) between January 2020 and March 2020. The study was approved by the Ethics Committee of the ‘Ärztekammer xxx’ (Medical Association xxx) in September 2019 (Eth-12/19) and conducted in accordance with the ethical standards of the Helsinki Declaration 1975.

The study was registered with the German clinical trial registry DRKS (DRKS00020539). No funding has been received.

The time of patient's hospital stay has been analyzed. The study is based on the patients' data available from their files.

The study was conducted in accordance to the STROCSS 2019 Guidelines [9].

### 2.1 Inclusion criteria

Patients who underwent elective laparoscopic IHR in TAPP technique and TAP block conduction were included.

### 2.2 Exclusion criteria

Patients who underwent a conversion to an open IHR, as well as patients undergoing primary open IHR were excluded. No individuals with femoral hernias were included.

Individuals with a lack of one-year-follow-up in the electronic file were excluded.

### 2.3 TAP block technique

The TAP block was performed either under direct visualization or with ultrasound imaging.

### 2.4 Direct visualization

The TAP block was performed under direct visualization using the laparoscope at the beginning of the operative procedure. While the abdominal cavity is insufflated, the surgeon palpated the lateral border of the rectus sheath to ensure adequate lateral placement. Then a 19 G needle was inserted percutaneously just above the iliac spine, inferior to the costal margin at the lateral end of the surgical field. By using the laparoscope to ensure that the peritoneum is not penetrated, the needle was advanced through the internal and external oblique and a small amount of local anesthetic was injected into the layer between the internal oblique and transversus abdominis. The appropriate dispersal along the layer was confirmed visually prior to the injection of the entire amount of local anesthetic. The same procedure was conducted on the contralateral side in case of a bilateral inguinal hernia (IH).

The amount of local anesthetic for unilateral tap block consisted of 75 mg ropivacaine mixed with 20 cc of normal saline. We used double the amount in a bilateral IH.

### 2.5 Ultrasound imaging

A TAP block conducted under anesthesia through ultrasound guidance (body habitus dictated linear high-frequency 12 MHz or curvilinear abdominal 6 MHz probe use) using a 22G needle and standard lateral approach was performed prior to the beginning of the operation.

### 2.6 Surgical procedure

The TAPP procedure has been performed by several different surgeons (n > 10). Next to an umbilical 10-mmport for the 30° telescope two 5-mm ports, 5 cm from umbilicus at the lateral borders of the rectus abdominis muscle were inserted. We insufflated CO2 to a pressure of 15 mmHg. The hernial sac was reduced. A Polypropylene mesh (Medtronic®) was inserted through the 10-mm port to cover the entire myopectineal orifice. The mesh was not fixated. While lowering the intra-abdominal pressure to 8 mm Hg, the peritoneum was closed by a running dissolvable non-self-locking suture.

### 2.7 Statistical analysis

Analysis was done using R (ver. 3.6.1). Data were presented as number (percentages) for nominal or mean ± SD/median (min-max) for metric variables. For the comparison of nominal variables between groups Fishers exact test was used, for metric variables normality was tested by using the Sapiro-Wilk test and hence the T-test or the Wilcoxon-test. A p-value < 0.05 was considered as statistically significant. No corrections for multiple testings were done. Using a two-sided two-sample *t*-test a power calculation has been performed.

In order to identify patients from the TAP group who benefit most from the therapy, as multivariate analysis we defined a decision tree regression model by using the R package “rpart".

## 3 Aims

The primary endpoint was the rate of CPIP revealed one year after surgery. CPIP was defined as pain impacting daily activities lasting at least 3 months postoperatively.

The secondary endpoints were relevant complications according to clavien-dindo-classification [10] during the hospital stay (CDC), operating time, length of hospital stay (LOS), pain at rest and exertion onr year after surgery, pain requiring treatment one year after surgery, one-year-recurrence rate, inguinal paresthesia.

### 3.1 Database

In October 2019, an MS Excel data sheet was provided. This data was imported into R (ver. 3.6.1), and multiple plausibility checks were performed. In November and December 2019, updates of this data were provided while inconsistencies were resolved.

### 3.2 Patient's selection

**Fig. 1:**
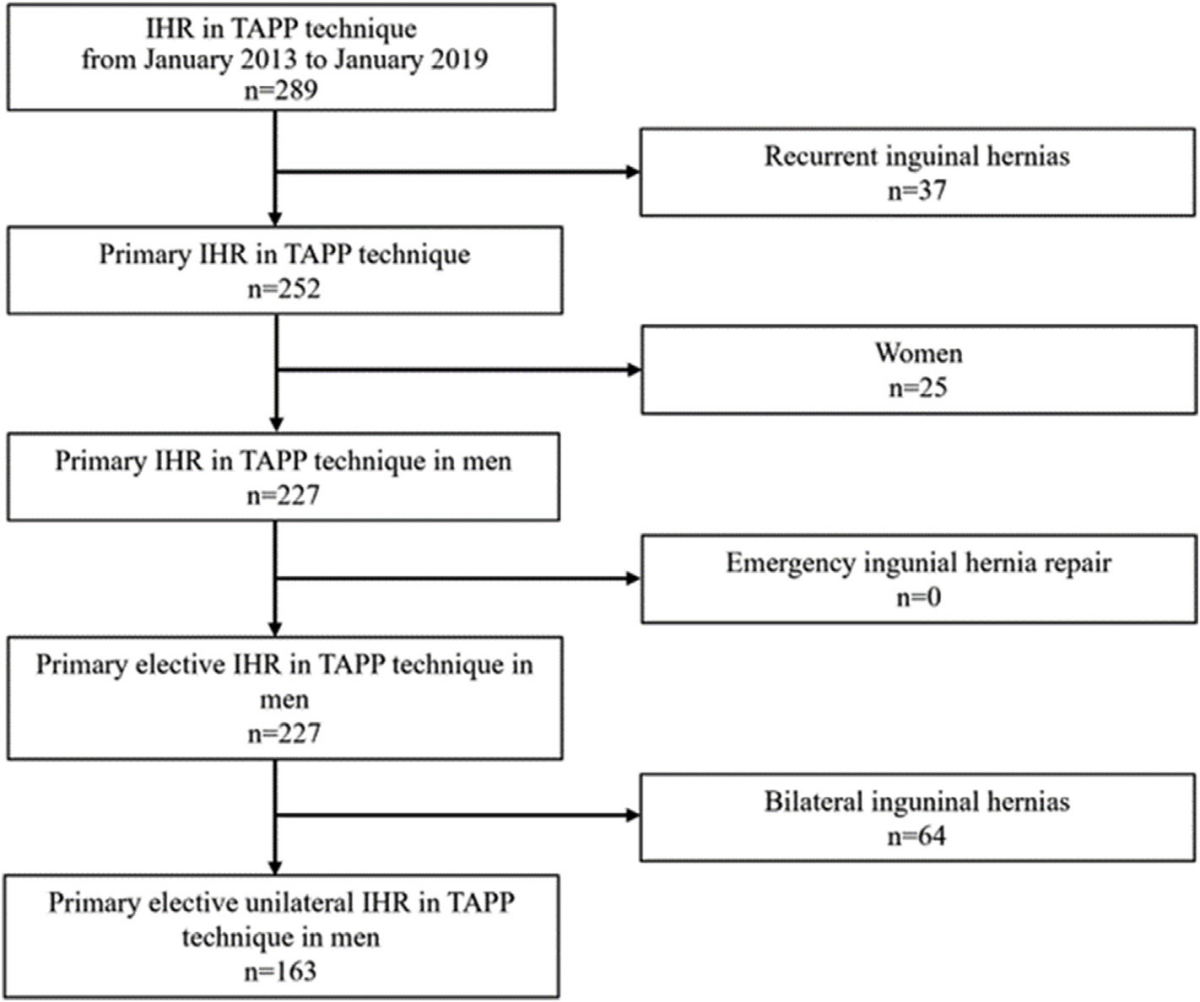
Flow chart on patients selection.

In order to compare the revealed data with published data from the German Hernia registry [11] a selection has been performed and depicts in Fig. 1.

Exclusion criteria were women, emergency surgery, femoral, bilateral and recurrent hernias.

## 4 Results

### 4.1 Biometric baseline characteristics

In this study 289 patients were enrolled (Table 1). The mean age was 59.93 (SD 15.29). 259 individuals were male and 30 female. 179 patients had an ASA I score, 102 an ASA II score and 8 an ASA III score. The average BMI was 25.72 kg/m^2^ (SD 2.16).

**Table 1:** Baseline characteristics.

Variable		Study group N=289
Age	*Years*	59.93 (SD 15.29)
Gender	*Male*	259
	*Female*	30
ASA preoperative	*I*	179
	*II*	102
	*III*	8
	*IV*	0
	*V*	0
BMI	Kg/m^2^	25.72 (SD 2.16)

ASA = American Society of Anesthesiologists physical status classification; BMI Body Mass Index continuous measurements are presented as mean (SD).

### 4.2 Perioperative baselines

The perioperative baselines are illustrated in Table 2. In terms of hernia location 208 suffered from an unilateral and 81 from a bilateral IH. Primary IH were diagnosed in 252 individuals whereas 37 patients were treated due to a recurrent IH (Table 2). Postoperative complications occurred in 18 patients (CD grade I, n = 14; CD grade II, n = 2; CD grade III, n = 3). Reasons for a reoperation were one bleeding, one early relapse and one urinary injury.

**Table 2:** Perioperative data.

Variable		Study group *n*= *289*
Hernia location		
	*unilateral*	208
	*bilateral*	81
Primary ingunial hernia		252
Recurrent inguinal hernia		37
Operating time	*Minutes*	54.77 (SD 22.04)
LOS	*Days*	2.12 (SD 0.65)
CDC Grading	*0*	93.42% (n=270)
	*I*	4.84% (n=14)
	*II*	0.25% (n=2)
	*II*	1% (n=3)
	*IV*	0% (n=0)
	*V*	0% (n=0)


CDC Clavien-dindo classification; Continous measurements are presented as mean (SD).

LOS Length of hospital stay; TAP transversus abdominis plane.

### 4.3 Primary and secondary endpoints

The endpoints one year after surgery are summarized in Table 3. 21 (7.2%) patients suffered from pain at rest, 26 (9%) from pain under exertion, 13 (4.5%) individuals from pain requiring treatment and 18 (6.2%) patients from CPIP. A hernia recurrence was yielded in 5 (1.73%) cases. 9 (3.1%) patients reported a seroma formation and 13 (4.5%) patients suffered from an inguinal dysesthesia. No trocar site hernia occurred (Table 3).

**Table 3:** One-year patient reported outcome.

Variable	Study group n= 289
Pain at rest	21 (7.2%)
Pain under physical exertion	26 (9.0%)
Pain requiring treatment	13 (4.5%)
CPIP	18 (6.2%)
Hernia relapse	5 (1.73%)
Seroma formation	9 (3.1%)
Dysaesthesia	13 (4.5%)
Trocar hernia	0

CPIP chronic postoperative inguinal pain.

### 4.4 Biometric and postoperative baselines in selected patients

The data from 163 patients were analyzed and compared with these from the German Herniamed Registry (n = 8799) [11] (Table 4). The average age was 59.85 years (SD 15.12). The mean BMI was 25.45 kg/m^2^ (SD 2.4). 92 (56.44%) individuals suffered from preoperative pain, 10 (6.13%) from pain at rest, 15 (9.2%) from pain under physical exertion, 4 (2.45%) from pain requiring treatment, 8 (4.9%) from chronic pain and 7 (4.29%) from ingunial dysaesthesia. In terms of pain under physical exertion, pain at rest and pain requiring treatment both groups did not statistical significant differ from each other.

**Table 4:** One-year patient reported outcome of male patients after primary, unilateral inguinal hernia repair in TAPP technique without mesh fixation.

Variable		TAP block group	Herniamed registry	p-value
		n= 163	n= 8.799	
Age	Years	59.85 (SD 15.12)	55.0 (SD15.6)	<0.001
BMI	Kg/m^2^	25.45 (SD 2.4)	25.9 (SD 3.3)	0.085
Preoperative pain		92 (56.44%)	5829 (66.25%)	0.186
Pain at rest		10 (6.13%)	466 (5.30%)	0.598
Pain under physical exertion		15 (9.2%)	884 (10.05%)	0.896
Pain requiring treatment		4 (2.45%)	260 (2.95%)	1.000
Chronic pain (3>month)	8 (4.9%)	NA		
Dysaesthesia	7 (4.29%)	NA		

BMI Body Mass Index; Continuous measurements are presented as mean (SD).

TAP Transversus abdominis plane.

### 4.5 Power calculation on primary endpoint: CPIP one year after surgery

To reveal differences regarding the CPIP one year after TAPP IHR with and without TAP block administration a group sample sizes of 334 (each) would achieve 90% power to detect a difference of 5% of CPIP between the null hypothesis that both group means CPIP rate is 5% and the alternative hypothesis that CPIP rate of group 2 is 10% with known group standard deviations and with a significance level (alpha) of 0,05 using a two-sided two-sample *t*-test.

## 5 Discussion

Chronic postoperative inguinal pain (CPIC) following inguinal hernia surgery is a major issue, which effects many patients [1,11]. The etiology is multifactorial. Perioperative nerve injuries or nerves that are being stucked and damaged by sutures or perforated by fixation devices such as tacks can lead to CPIP [12]. Moreover, nerval structures can also be affected in cases of mesh shrinking. In general CPIP can be caused by these neuropathic pain (damages to nerval structures) and/or nociceptive pain (caused by the release of metabolic substances due to tissue damage or damage to organs) [1,2,13,14].

Due to different definitions, different assessment times and measurement methods the incidence of CPIP after open and laparoscopic IHR is heterogeneous reported in literature. It ranges from 0.7 to 43.3% [2]. According to the International guidelines for prevention and management of post-operative chronic pain following inguinal hernia surgery a prevalence of 0.5–6.0% following open and laparoscopic IHR repair has been estimated [2,3].

Early postoperative pain has been described as one risk factor for CPIP [1,2,15]. To that Callesen et al. (1999) conducted a prospective consecutive case series study by questionnaire of 466 unselected adult patients one year after open IHR. The authors stated, that CPIP can be predicted by the intensity of early postoperative pain [16]. Also Aasvang et al. (2014) identified early postoperative pain as a risk factor for CPIP, when conducting a multivariate analysis among 464 patients undergoing open and laparoscopic transabdominal preperitoneal elective IHR [13].

We recently revealed the positive effect of the TAP block on postoperative pain and cumulative analgesic need medication after TAPP IHR (n = 766). The individuals, who received a TAP block reported a reduced VAS-Score in the post-anesthesia care unit (Control group with oral/intravenous opioid and non-opioid medication, n = 402, 1.14 (1.37); TAP block group, n = 364, 0.75 (1.25); p < 0.001) following TAPP IHR [5]. This led to our hypothesis, that the TAP block, as an early potent non-opioid postoperative pain killer, may also sufficiently reduce CPIP following TAPP repair. Although CPIP more often occurs after open hernia repair in up to 15% of cases patients suffer from chronic pain following TAPP procedure [11,17].

We compared our results in terms of pain at rest, pain under physical exertion and pain requiring treatment with those from the Herniamed Registry. The patients were selected (unilateral primary inguinal hernia among men; Table 4). Although the rate of pain under physical exertion and pain requiring treatment was lower among our analyzed patients, the differences were not statistically significant (Table 4). On the other hand, it is most likely that a certain amount of the 8.799 reported patients from the Herniamed Registry received a TAP-block. The Registry is not questioning local anesthetic determination. Moreover we revealed, when conducting a survey among surgical departments in 2019, that nearly 50% of these regularly performs a TAP-block. Therefore, it is imaginable that among the registered patients, who did not receive a TAP block, the rate of pain at rest, pain under physical exertion and pain requiring treatment might be even higher, We further compared our results with published data consisting of individuals, who underwent primary TAPP IHR without mesh clip fixation and without local analgesic determination. Our study at hand revealed a 4.9% CPIP rate among selected patients (Table 4) and a 6.2% overall CPIP rate (Table 3). Bansal et al. (2013) prospective analyzed 154 patients. The authors published a CPIP of only 2% after 12 months [18]. But patients with significant comorbidities and previous abdominal surgical interventions were excluded. Only 0.5% of CPIP was reported by Li et al. (2017). The authors conducted a randomized clinical trial among 100 patients. But next to significant comorbidities and previous abdominal operations even scrotal hernias and a defect size of a hernia ring more than 4 cm were exclusion criteria's [19]. Higher CPIP rates in comparison to our results were also published. To that Dickinson et al. (2008) revealed a CPIP rate of 13%. The median interval since surgery was 5.0 years (range 1.18–9.53) [20], but no statement about mesh fixation was given. In addition Bittner et al. (2010) published a CPIP rate of 15.9% 6 month after TAPP IHR when conducting a prospective trial among 276 individuals [21].

In general, the published data on that topic are hardly comparable due to different used definitions for chronic pain and follow-up periods. A sample size of 334 (each) would be needed to confirm our hypothesis that the TAP block leads to a reduction of CPIP one year after IHR in TAPP technique [20,21].

It has been reported by several authors, that the TAP block sufficiently reduced early postoperative pain and cumulative analgesic need medication following laparoscopic IHR [5,[22], [23], [24]]. Another argument to further implement the TAP block into daily routine may be, based on our findings, the potency of CPIP rate reduction. Hence, prospective clinical trials using chronic pain definitions according to international guidelines are crucial to evaluate our hypothesis [1,2].

Naturally when patients are suffering less from chronic pain, it likely will lead to an overall reduced opioid consumption. Severe opioid side effects such as nausea, vomiting, constipation and especially the addiction potential with increasing numbers of opioid related deaths could be prohibited [1,25].

As limitations the retrospective study design and the small sample size have to be mentioned. Moreover, several surgeons performed the IHR. In addition, the TAP-block has been administrated ultrasound-guided preoperatively and visual guided intraoperatively. Although recent literature did not indicate differences in terms of analgesic potency it might be an issue. Further trial should focus on one decent approach. Due to a lack of documentation in patients files we were unable to clearly identify ultrasound-guided preoperatively and visual guided intraoperatively TAP block administration.

## 6 Conclusion

Due to our findings we assume that the TAP block may reduce CPIP, postoperative pain under physical exertion and pain requiring treatment following IHR in TAPP technique. The TAP block should be further implemented into daily routine. Further randomized clinical trials are mandatory to evaluate the hypothesis.

## Ethical approval

Ethical approval has been received.

## Sources of funding

All authors have no source of funding.

## Author contribution

CP (corresponding author): Contribution to the paper: author, data collection, data analysis and interpretation, writing the paper, examination and treatment of patients.

GD (co-author): Contribution to the paper: data extraction.

SA (co-author): Contribution to the paper: data extraction.

SA (co-author): Contribution to the paper: data analysis, treatment of patients.

JF (co-author): Contribution to the paper: data extraction.

KB (co-author): Contribution to the paper: data analysis, grammatical correction.

UG (co-author): Contribution to the paper: data analysis.

MS (senior-author): Contribution to the paper: data analysis, examination and treatment of patients.

## Research data for this article

Due to the sensitive nature of the revealed data in this study, they will not be shared.

The data that has been used is confidential.

## Provenance and peer review

Not commissioned, Editor reviewed.

## Declaration of competing interest

None.

## Appendix A Supplementary data

Application Application
